# Cutis laxa congénital: à propos d’un cas

**DOI:** 10.11604/pamj.2019.34.195.17110

**Published:** 2019-12-12

**Authors:** Aziza El Ouali, Manal Azizi, Siham Dikhaye, Noufissa Benajiba

**Affiliations:** 1 Service de Pédiatrie, CHU Mohammed VI, Université Mohammed I, Oujda, Maroc; 2 Service de Dermatologie CHU Mohammed VI, Université Mohammed I, Oujda, Maroc

**Keywords:** Cutis laxa, tissu conjonctif, Cutis laxa, connective tissue

## Abstract

Les « cutis laxa » (CL) sont des affections rares du tissu élastique, caractérisées par une hyperlaxité cutanée. Elles peuvent être congénitales ou acquises. Les formes héréditaires constituent un groupe hétérogène par la gravité de leurs atteintes viscérales et leur mode de transmission. Trois groupes ont été individualisés sur la base de la transmission génétique: autosomique dominante, autosomique récessive et récessive liée à l'X. La sévérité des atteintes viscérales conditionne le pronostic des CL héréditaires qui peut être fatal à brève échéance en cas d'atteinte cardiaque ou pulmonaire. L'objectif de notre travail est de rappeler aux praticiens cette affection rarissime, ceci à travers l'observation d'un nourrisson suivi depuis son seizième jour de vie pour une détresse respiratoire.

## Abstract (en)

"Cutis laxa" (CL) are rare elastic tissue disorders characterized by loose, sagging skin. They can be a congenital or acquired condition. Inherited cutis laxa is a heterogeneous group of disorders characterized by the severity of their visceral involvement and by their mode of transmission. Three groups have been identified on the basis of their genetic transmission: autosomal dominant, recessive autosomal, X-linked recessive. The severity of the visceral involvement affects the prognosis of inherited CL which is potentially fatal in the short term in patients with cardiac or pulmonary involvement. This study aims to remind clinicians of this rare affection through direct observation of an infant being followed-up for respiratory distress sixteenth days after birth.

## Introduction

Les « cutis laxa » (CL) sont des affections relativement rares, caractérisées par un aspect de sénescence précoce, d'hyper laxité cutanée associée à des manifestations systémiques variables. Elles peuvent être congénitales ou acquises [1]. Les formes héréditaires peuvent être transmises sous diverses formes: autosomiques, dominantes ou récessives et liées à l'X. Seuls quelques rares cas; environ 200 familles; ont été rapportés à ce jour dans la littérature nous en décrivons un nouveau cas.

## Patient et observation

**Figure 1:**
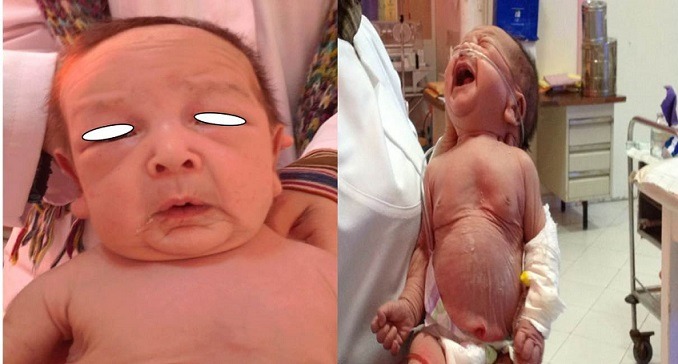
Image de peau ridée, abondante, pendante conférant un aspect vieilli

**Figure 2:**
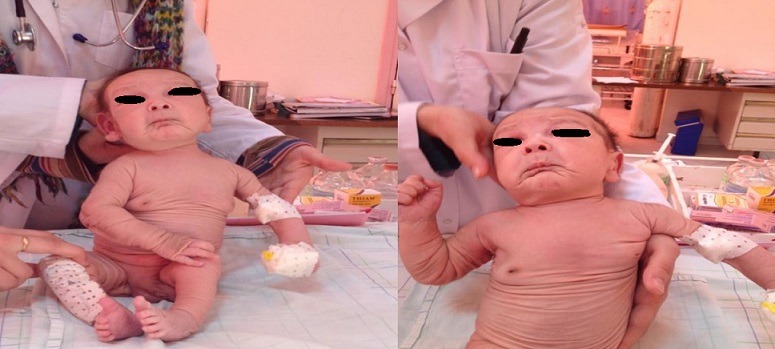
Un excès cutané touchant tout le corps avec de nombreux replis flasques avec laxité ligamentaire

Nous rapportons l'observation d'un nouveau-né de sexe masculin, issu de parents consanguins de 1^er^ degré et indemne de toute affection cutanée. La mère était 2^ème^ geste et 2^ème^ pare. Il n'y avait pas de cas similaires dans la famille. L'enfant était issu d'une grossesse de déroulement normal et menée à terme avec un accouchement par voie basse; il a été admis à J16 de vie dans un tableau de détresse respiratoire. A l'examen, le nouveau-né était eutrophique (PN: 4Kg230, taille: 53cm, PC: 38cm), apyrétique, ayant une voix rauque. L'examen cutanéo-muqueux avait objectivé une peau ridée, abondante, pendante conférant un aspect vieilli, avec de nombreux replis flasques et un excès cutané touchant tout le corps (Figure 1, Figure 2). On notait également une laxité ligamentaire et des hernies inguinales bilatérales. L'examen pleuropulmonaire trouvait un nouveau-né polypnéique à 72 cycles/min, avec un tympanisme du côté gauche et un score de Silverman à 5. Une infection néonatale a été écartée aussi bien cliniquement, biologiquement que radiologiquement. La recherche d'une autre atteinte viscérale et des malformations s'est révélée négative. Le diagnostic de « cutis laxa », fortement évoqué, a été confirmé par l'examen histologique d'une biopsie cutanée. L'évolution a été marquée par l'aggravation de la détresse respiratoire. La radiographie thoracique était en faveur d'un pneumothorax gauche ayant nécessité une exsufflation avec une bonne évolution clinique. Le patient a été réadmis une semaine plus tard dans un tableau plus bruyant. La radiographie thoracique était en faveur d'un emphysème pulmonaire qui a conduit une semaine plus tard au décès de l'enfant.

## Discussion

Les «cutis laxa» (CL) désignent des affections rares, héréditaires ou acquises du tissu élastique. Plusieurs synonymes désignent cette maladie dans la littérature médicale (élastolyse, chalazodermie, dermatolysie, dermatochalasis) mais le terme cutis laxa reste le plus utilisé. Sur le plan clinique, elles ont en commun un état d'inélasticité et de relâchement cutané; ainsi la peau parait élastique, lâche et pendante produisant un aspect froissé et vieilli [2, 3]. Ce qui était le cas pour notre patient. L'examen histologique ou ultra structural montre une diminution et une désorganisation du réseau des fibres élastiques du derme. Les bases moléculaires de ces altérations demeurent inconnues dans la plupart des cas. Dans certains, des mutations de protéines impliquées dans l'organisation de l'architecture des fibres élastiques ont été mises en évidence [4, 5]. Ces mutations entraînent des anomalies de synthèse de l'élastine ou des défauts structuraux des protéines de la matrice extracellulaire. Les formes héréditaires sont hétérogènes par la gravité de leurs atteintes viscérales; qui conditionnent le pronostic vital et par leur mode de transmission. Elles sont transmises sous différents formes: autosomique dominante, autosomique récessive et récessive liée à l'X.

**«Cutis laxa» autosomiques dominantes:** dans les CL autosomiques dominantes, les manifestations cutanées sont généralement présentes dès la naissance ou au cours de la petite enfance. Les manifestations viscérales sont absentes ou modérées comportant des lésions pulmonaires (emphysème, sténose de l'artère pulmonaire, bronchectasies), cardiaques (régurgitation valvulaire mitrale ou tricuspidienne, anévrisme de l'aorte, hypertrophie ventriculaire droite), des hernies digestives et prolapsus génitaux [6, 7]. Ces formes héréditaires sont associées à des mutations du gène ELN, ou du gène de la fibuline-5 (FBLN5) [8, 9].

**«Cutis laxa» liée à l'X:** cette variété de CL; connue sous le nom de syndrome des cornes occipitales; est identique à l'ancien syndrome d'Elhers-Danlos de type IX et à une variante de bon pronostic de la maladie de Menkes. Cliniquement, hormis l'atteinte cutanée, il existe une dysmorphie faciale avec des exostoses occipitales formant des cornes, des carotides sinueuses et sténoses artérielles intracrâniennes, des sténoses et diverticules du tractus urinaire, une hyperlaxité ligamentaire, un retard mental et un retard de croissance. Elle est associée à des mutations du gène ATP7A codant pour un transporteur du cuivre et justifie une étude du métabolisme du cuivre (dosage de la céruléoplasmine et de la cuprémie).

**«Cutis laxa» autosomique récessive:** il est associé à des manifestations cliniques sévères. Les enfants atteints de la maladie (souvent avec les parents consanguins) ont la peau lâche évidemment à la naissance, et la plupart meurent au cours de la petite enfance de complications cardio-pulmonaires, bien que d'autres systèmes puissent être affectés [10]. On en distingue 3 formes. Toutefois en raison du manque du laboratoire spécifique et de l'évolution rapidement fatale, l'étude génétique n'a pas été réalisée chez notre patient. Mais nous suggérons qu'il avait une forme autosomique récessive devant la notion de consanguinité parentale, l'atteinte cutanée précoce dès la naissance et l'évolution rapidement fatale par emphysème pulmonaire [11, 12].

## Conclusion

Le «cutis laxa» est une affection rare dont la présentation clinique et le mode de transmission montrent une hétérogénéité considérable, hormis le préjudice esthétique important qu'elle entraine, certaines atteintes systémiques conditionnent le pronostic vital à brève échéance. La prise en charge d'un patient atteint de cutis laxa doit être pluridisciplinaire, sans qu'aucun traitement n'ait à ce jour fait preuve d'efficacité. D'autre part l'identification du mode de transmission qui, lorsqu'elle est possible, peut permettre un conseil génétique.

## Conflits d’intérêts

Les auteurs ne déclarent aucun conflit d'intérêts.
